# A composite scaffold of MSC affinity peptide-modified demineralized bone matrix particles and chitosan hydrogel for cartilage regeneration

**DOI:** 10.1038/srep17802

**Published:** 2015-12-03

**Authors:** Qingyang Meng, Zhentao Man, Linghui Dai, Hongjie Huang, Xin Zhang, Xiaoqing Hu, Zhenxing Shao, Jingxian Zhu, Jiying Zhang, Xin Fu, Xiaoning Duan, Yingfang Ao

**Affiliations:** 1 Institute of Sports Medicine, Beijing Key Laboratory of Sports Injuries, Peking University Third Hospital, 49 North Garden Road, Haidian District, Beijing 100191, PR China

## Abstract

Articular cartilage injury is still a significant challenge because of the poor intrinsic healing potential of cartilage. Stem cell-based tissue engineering is a promising technique for cartilage repair. As cartilage defects are usually irregular in clinical settings, scaffolds with moldability that can fill any shape of cartilage defects and closely integrate with the host cartilage are desirable. In this study, we constructed a composite scaffold combining mesenchymal stem cells (MSCs) E7 affinity peptide-modified demineralized bone matrix (DBM) particles and chitosan (CS) hydrogel for cartilage engineering. This solid-supported composite scaffold exhibited appropriate porosity, which provided a 3D microenvironment that supports cell adhesion and proliferation. Cell proliferation and DNA content analysis indicated that the DBM-E7/CS scaffold promoted better rat bone marrow-derived MSCs (BMMSCs) survival than the CS or DBM/CS groups. Meanwhile, the DBM-E7/CS scaffold increased matrix production and improved chondrogenic differentiation ability of BMMSCs *in vitro*. Furthermore, after implantation *in vivo* for four weeks, compared to those in control groups, the regenerated issue in the DBM-E7/CS group exhibited translucent and superior cartilage-like structures, as indicated by gross observation, histological examination, and assessment of matrix staining. Overall, the functional composite scaffold of DBM-E7/CS is a promising option for repairing irregularly shaped cartilage defects.

Articular cartilage is a well-organized tissue that possesses excellent biomechanical properties, such as low friction and compressive and tensile properties. It plays an important role in the movement and lubrication of synovial joints. Once damaged or diseased, articular cartilage is challenging to repair or reconstruct because of its poor intrinsic healing potential^1^,^2^. Ideally cartilage defects should be repaired with tissue that has appropriate structure, composition, and mechanical properties to restore joint function and prevent additional deterioration of the joint^3^. Although many attempts have been conducted to address this problem, most of the current treatment modalities were insufficient to regenerate functional cartilage similar to the native articular cartilage^4^. Stem cell-based tissue engineering manipulates endogenous stem cells, scaffolds, and biological agents to enhance the natural capacity of the body to self-repair by providing a microenvironment for tissue development and regeneration, and it is a promising technique for cartilage repair^5^,^6^. Bone marrow-derived mesenchymal stem cells (BMMSCs) have been widely used in cartilage tissue engineering because of their significant chondrogenic potential^7^.

Scaffold is one of the three key elements for tissue engineering; and the functional modification of scaffolds has been a focus of research in cartilage regeneration for the past decades^8^,^9^. Compared with synthetic material scaffold, natural material scaffold is gaining increasing interest because of its excellent biocompatibility and biodegradability without toxic by-products^10^,^11^. Chitosan (CS) hydrogel is a typical natural material with significant advantages in cartilage tissue engineering because of its structural similarity to sulfated glycosaminoglycan (GAG), providing a friendly microenvironment for chondrocyte proliferation and extracellular matrix (ECM) production, maintaining the correct phenotype, and sustaining chondrogenesis^12^,^13^,^14^. However, inadequate mechanical stability of the CS scaffold restricts its application in clinical. To address this problem, solid-supported CS hydrogel scaffold has been constructed by combining CS hydrogel and solid-state biomatrix, thereby significantly improving its mechanical stability^15^. In previous study, we constructed a solid-supported scaffold comprising CS thermogel and demineralized bone matrix (DBM) cylinders for cartilage regeneration^16^. Results showed that this solid-supported scaffold platform can retain more cells while at the same time provide sufficient strength for cartilage tissue engineering, and this platform is suitable for proliferation and chondrogenesis of BMMSCs *in vitro* and *in vivo*.

In addition to maintaining adequate mechanical properties, functional modification of the scaffold with endogenous MSC homing ability is also important for stem cell-based cartilage regeneration strategy as MSCs occur in low quantity in the bone marrow but many BMMSCs are needed^17^,^18^. Cell-adhesive ligands or affinity peptides provide a more effective method for cell recruitment of biomaterial scaffolds^19^,^20^. Arginine-glycine-aspartic acid is a well-known cell-adhesive peptide that is widely applied in material modification derived from fibronectin in the ECM. However, this peptide is non-specific because fibronectin exists in all cell types^21^,^22^. To promote BMMSCs recruitment with high specificity and efficiency, we identified an affinity peptide sequence named E7 using phage display technology and successfully applied it in different scaffolds *in vitro* or *in vivo* without species specificity^16^,^23^,^24^.

As cartilage defects are usually irregular with various shapes in clinical, scaffolds that can be easily molded to fill any shape of cartilage defects and closely integrate with the host cartilage are desirable^25^. However, most of the currently available biomaterial scaffolds, except for liquid biomaterials, have poor moldability and cannot fully fill the irregularly shaped defects. Any gaps between the scaffold and the host cartilage might be adverse for cartilage regeneration because the poor biomechanical properties of the gaps can restrict cell adhesion and proliferation^26^. Scaffolds of liquid biomaterials, though with high moldability, might have insufficient mechanical strength. In the current work, we designed a composite scaffold combining E7-modified DBM (DBM-E7) particles and CS hydrogel for stem cell-based cartilage tissue engineering, in an attempt to integrate a moldable hydrogel and a functional biomaterial unit into one 3D scaffold for cartilage regeneration. In this scaffold, the DBM-E7 particles play a role in improving biomechanical properties and MSCs homing, while the CS provides a friendly 3D cell-support microenvironment and maintains the integrity of scaffold. Pure CS scaffolds (CS group) and composite scaffolds of DBM and CS (DBM/CS group) were also established as controls (Fig. 1A). To improve the moldability of the scaffold, a whole piece of DBM was first ground to particles, ranging in diameter from 100 μm to 800 μm, and then blended in the CS hydrogel. The surface-to-volume ratio of the DBM particles increased compared with that of the whole DBM piece, promoting the E7 conjugation rate. This functional composite DBM-E7/CS scaffold was supposed to enrich BMMSCs homing, provide suitable mechanical properties and cell-support system, and sustain chondrogenic properties *in vitro* and *in vivo*. To further investigate the feasibility of this hypothesis, the properties and function of the DBM-E7/CS scaffold were comprehensively studied.

## Results

### Conjugation of E7 peptide to DBM

**Figure 1:**
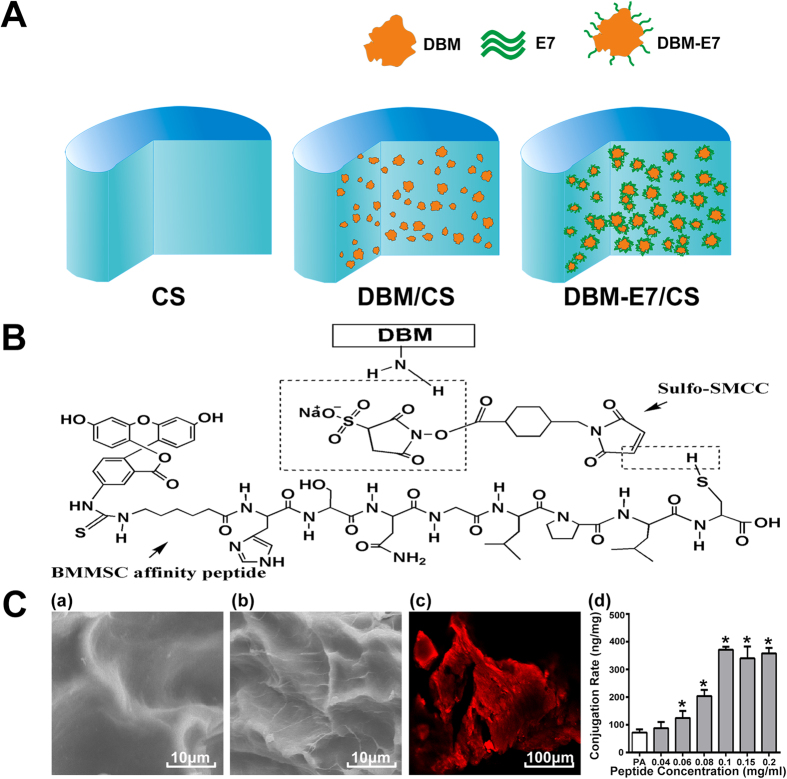
Schematic illustration of the three different structural scaffolds, the conjugating process of E7 peptide to DBM particles and characterization of DBM-E7 particles. (**A**) CS scaffold is composed of pure CS hydrogel, DBM/CS scaffold is a mixture of CS hydrogel and DBM particles, and DBM-E7/CS scaffold is composed of DBM-E7 particles blended in CS hydrogel. (**B**) BMMSCs affinity peptide was covalently conjugated with DBM via cross-linker of sulfo-SMCC. SEM images of representative areas of (**C** a) DBM and (**C** b) DBM-E7 particles. (**C** c) Confocal scanning of DBM-E7 particle with red fluorescence of rhodamine. (**C** d) Quantification of the amount of peptide conjugated to scaffolds (PA, physical adsorption; *p < 0.05 vs. PA).

The E7 peptide was successfully covalently conjugated to the DBM particles (Fig. 1B). To determine the characteristics of DBM-E7 particles, scanning electron microscopy (SEM) and confocal microscopy were conducted to determine the characteristics of the DBM-E7 particles. SEM revealed that different from the surface of DBM particles (Fig. 1C a), the surface of DBM-E7 particles became rough with a thin layer of peptide materials after E7 peptide conjugation, which may facilitate specific BMMSCs recruitment (Fig. 1C b). Confocal scanning microscopy images showed that DBM-E7 particles exhibited red fluorescence when the E7 peptide was labeled by rhodamine (Fig. 1C c). Covalent conjugation led to a significantly higher density of E7 peptide on the surface of DBM particles than the physical adsorption (PA) (Fig. 1C d). The concentration of E7 peptide conjugated to DBM particles increased as the concentration of E7 peptide-conjugating solution increased up to 0.1 mg mL^−1^, beyond which additional E7 peptide did not improve the conjugation rate. Therefore, the E7 concentration of 0.1 mg mL^−1^ was used for conjugation in all subsequent experiments. These results demonstrated that conjugation of E7 peptides to DBM particles using a heterobifunctional cross-linker of sulfosuccinimidyl-4-(N-maleimidomethyl) cyclohexane-1-carboxylate (sulfo-SMCC) was sufficient and stable. In this way, DBM-E7 particles were established.

### Characterization of three scaffold groups

**Figure 2:**
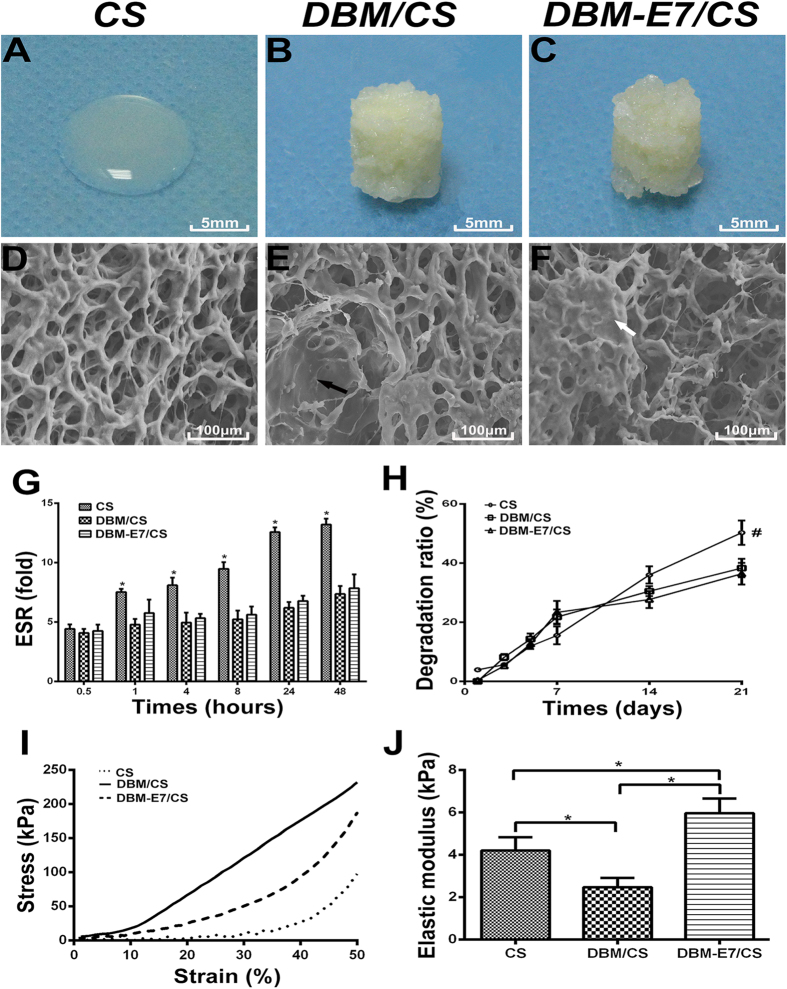
Characterization of the three different structural scaffolds. (**A–C**) Gross morphologies of CS, DBM/CS, and DBM-E7/CS scaffolds. (**D–F**) SEM images of the three scaffold groups (black arrow: DBM particles; white arrow: DBM-E7 particles). (**G**) ESR of the three scaffold groups (*n* = 5, *p < 0.05). (**H**) Degradation ratio of the three scaffold groups (*n* = 5, *p < 0.05). (**I**) Stress–strain curves of the three scaffold groups (*n* = 3, *p < 0.05). (**J**) Elastic modulus of the three scaffold groups (*n* = 3, *p < 0.05).

Pure CS scaffolds exhibited a semitransparent gel structure, whereas DBM/CS and DBM-E7/CS scaffolds showed a solid-support structure and can be easily shaped to columnar state (Fig. 2A–C). All of the three scaffolds are moldable and could be used to repair irregularly shaped cartilage defects. SEM observation showed that the pore size of the CS gel ranged from 30 μm to 80 μm, with no significant changes after the addition of DBM or DBM-E7 particles. The porosities of CS, DBM/CS, and DBM-E7/CS scaffolds were 72% ± 7.2%, 69% ± 5.4%, and 68% ± 6.8%, respectively, with no significant differences among the three groups (Fig. 2D–F, *n* = 5 in each group, p > 0.05).

In the equilibrium swelling ratio (ESR) test, all types of scaffolds gradually absorbed water. However, the ESR results of the CS scaffold were significantly higher than that of the DBM/CS and DBM-E7/CS scaffold at different time intervals, except at 0.5 h. The ESR results between the DBM/CS and DBM-E7/CS scaffold showed no significant difference (Fig. 2G). The degradation test showed that the CS, DBM/CS, and DBM-E7/CS scaffold degraded at similar rates during the early periods of incubation (1, 3, and 5 days). The degradation ratio of the DBM/CS and DBM-E7/CS scaffold became higher than that of the CS scaffold at day 7, and then became significantly lower than those of the CS group at days 14 and 21 (Fig. 2H).

The stress–strain curves of the three different scaffolds in the biomechanical test showed that the CS scaffold had minimal stress forces during 0%–30% strain of scaffolds, and then demonstrated a linear increase of stress between 40% and 50% strains. The DBM-E7/CS group presented a gradual increase in stress from 20% to 50% strain with weaker strength than the DBM/CS group but greater strength than the CS group. The DBM/CS group exhibited a linear increase in stress from 20% to 50% strain (Fig. 2I). The elastic moduli of DBM-E7/CS scaffolds were significantly higher than those of the CS and DBM/CS groups, which were determined from the slope of the linear portion of the stress–strain curve (Fig. 2J).

### BMMSCs characterization

**Figure 3:**
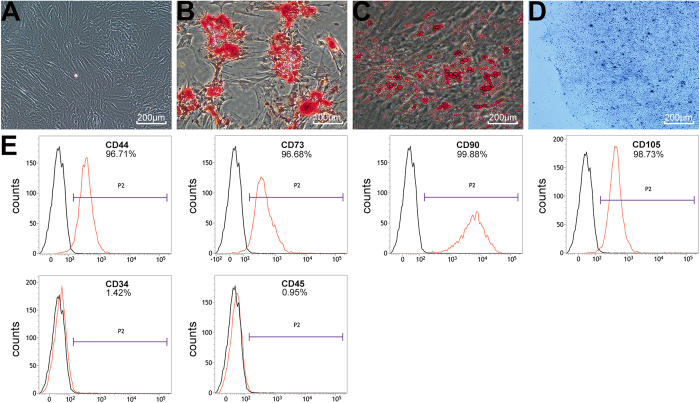
Tri-lineage differentiation potentials and flow cytometry analysis of BMMSCs. (**A**) Homogeneous distributions were observed on BMMSCs of P3. (**B**) Osteogenesis was examined using alizarin red staining. (**C**) Adipogenic capacity was verified using oil red O staining. (**D**) Chondrogenic potential was assessed by toluidine blue staining. (**E**) The results of flow cytometry showed that BMMSCs of P3 had a specific phenotype.

BMMSCs at passage three (P3) exhibited fusiform morphology and homogeneous distribution after passage and expansion (Fig. 3A). The tri-lineage differentiation experiment showed that BMMSCs had the potential to differentiate into osteoblasts (Fig. 3B), adipocytes (Fig. 3C), and chondrocytes (Fig. 3D). The results of flow cytometry analysis demonstrated that BMMSCs had homogeneous phenotype after isolation and expansion. Positive phenotypic markers, CD44 (96.71%), CD73 (96.68%), CD90 (99.88%), and CD105 (98.73%), were overexpressed on BMMSCs. The lipopolysaccharide receptor CD34 (1.42%) and the leukocyte common antigen CD45 (0.95%) were negatively expressed (Fig. 3E).

### Morphology and distribution of BMMSCs grown on scaffolds

**Figure 4:**
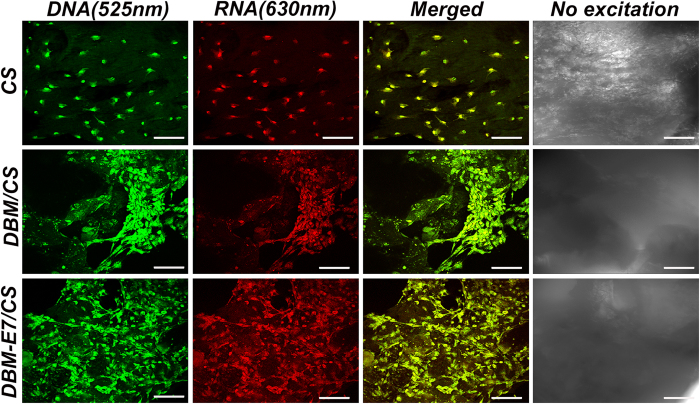
Morphology, adhesion, and distribution of BMMSCs grown on the three scaffold groups. BMMSCs grown on the three scaffold groups were uniformly stained with green and red fluorescence. Green: intercalated DNA by AO with fluorescence at 525 nm. Red: electrostatic RNA by AO with fluorescence at 630 nm. Yellow: combination of green and red above. No excitation: the bright field. Scale bar = 100 μm. Magnification: ×25.

**Figure 5:**
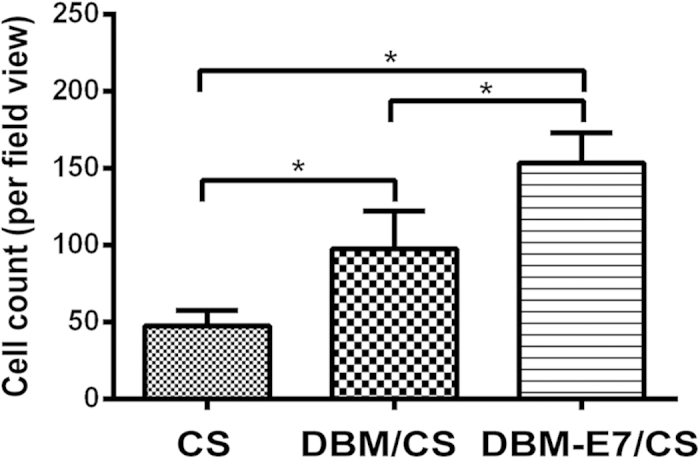
The BMMSCs number on the three scaffold groups at 24 hours (*p < 0.05).

Confocal microscopy was used to assess the morphology and distribution of BMMSCs grown on scaffolds (Fig. 4). After 24 hours of culture, BMMSCs grew well on CS, DBM/CS, and DBM-E7/CS scaffolds, showing typical fusiform BMMSC morphology according to confocal laser microscopic images. The DNA and RNA of BMMSCs interacted with acridine orange (AO) (515–545 nm for DNA and 590–630 nm for RNA). The nuclei of BMMSCs were stained green or yellow-green, indicating living normal cell. All BMMSCs were uniformly stained with red fluorescence, suggesting that the cytoplasm of the BMMSCs was uniform with no ruptured or apoptotic cells. The BMMSCs exhibited a 3D and homogeneous distribution on CS scaffolds, but a maldistribution in the gap between the DBM particles and the CS part on DBM/CS scaffolds. For DBM-E7/CS scaffolds, BMMSCs showed a significant cluster on the DBM-E7 particles because of its specific BMMSCs homing capacity. Image-Pro Plus (IPP) 6.0 software was used to evaluate the BMMSCs number on the three groups at 24 hours (Fig. 5). The result showed DBM-E7/CS scaffold significantly increased the number of cells that were grown on the scaffold 24 hours after seeded.

### BMMSCs proliferation and cartilage matrix production *in vitro*

**Figure 6:**
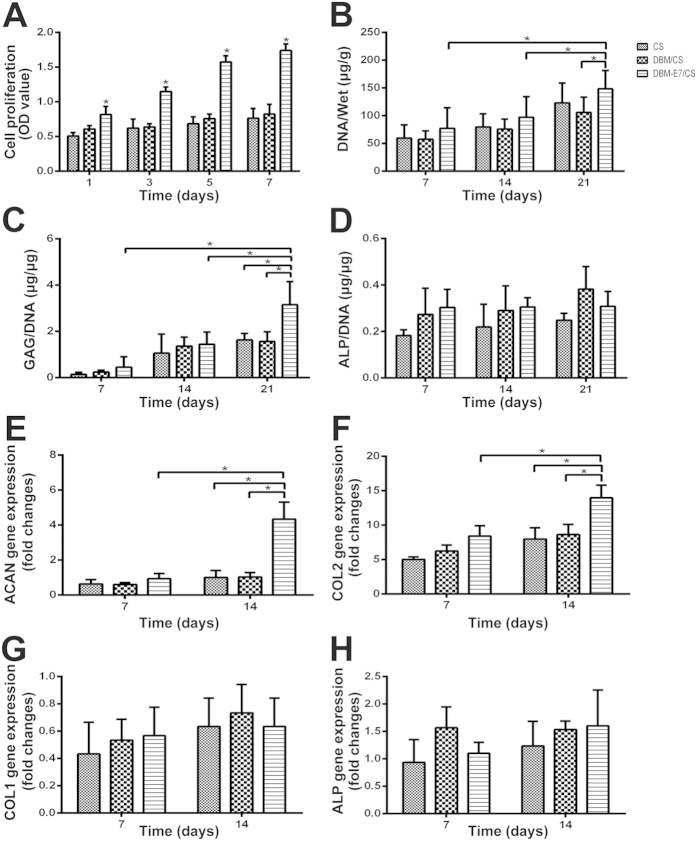
Proliferation, cartilage matrix production and chondrogenic differentiation of BMMSCs on different scaffolds *in vitro*. (**A**) OD values of CCK-8 assay of BMMSCs cultured in different scaffolds (*n* = 3, *p < 0.05). (**B**) DNA content of BMMSCs for the three scaffold groups (*n* = 3, *p < 0.05). (**C**) Sulfated GAG production of the three scaffold groups analyzed using DMMB assay (*n* = 3, *p < 0.05). (**D**) ALP products deposited in CS, DBM/CS, and DBM-E7/CS scaffolds were determined by ELISA assay (*n* = 3, p > 0.05). (E-H) The expression of hyaline cartilage-specific genes, (**E**) ACAN and (**F**) COL2, and osteogenic genes, (**G**) COL1 and (**H**) ALP (*n* = 3, *p < 0.05).

The results of CCK-8 assay (Fig. 6A) showed that the proliferative capacity of BMMSCs on all types of scaffolds increased with culture time. At each time point, the optical density (OD) values of the DBM-E7/CS group were significantly higher than those of the CS and DBM/CS groups, with no difference between the CS and DBM/CS groups. The content and weight of DNA in the DBM-E7/CS group increased significantly over time during BMMSCs culture *in vitro*, and they were significantly higher than those of the DBM/CS group at 21 days (Fig. 6B). Similar to the results of DNA, the GAG/DNA value on the DBM-E7/CS scaffold also increased significantly as the time of cell culture extended and was significantly higher than those of CS and DBM/CS scaffolds at 21 days (Fig. 6C). These results indicated that the DBM-E7/CS scaffold had excellent ability to sustain BMMSCs recruiting, cell proliferation, and GAG production. There was no difference in ALP/DNA values between groups or among different time points (Fig. 6D).

### Chondrogenic differentiation of BMMSCs on scaffolds *in vitro*

The hyaline cartilage-specific markers (ACAN and COL2) and osteogenesis markers (COL1 and ALP) were tested for the scaffolds’ ability to promote BMMSCs chondrogenic differentiation or dedifferentiation based on the results of RT-PCR (Fig. 6E–H). Cartilage-specific genes, ACAN and COL2, showed greater expression on the DBM-E7/CS scaffold than on the CS or DBM/CS scaffolds. The gene expression levels of COL1 and ALP, however, had no significant difference between the three groups at each time point. These results suggested that DBM-E7/CS scaffolds could promote BMMSCs to differentiate to chondrocytes without osteogenesis.

### Chondrogenesis of scaffolds *in vivo*

**Figure 7:**
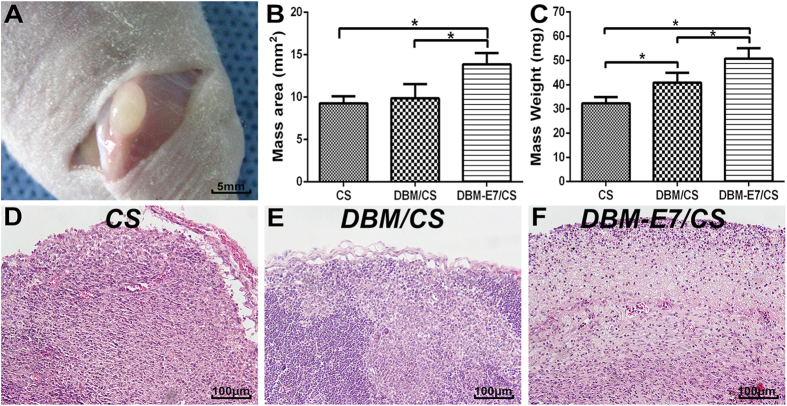
General evaluation of the neocartilaginous tissue of the three groups. (**A**) Gross observation of hyaline cartilage-like tissue. (**B**) Mass area analysis of regenerated neotissue (*n* = 5, *p < 0.05). (**C**) Mass weight of tissue regenerated by the three scaffolds after harvesting (*n* = 5, *p < 0.05). (**D–F**) H&E staining of the neo-tissue generated by CS, DBM/CS, and DBM-E7/CS scaffolds.

**Figure 8:**
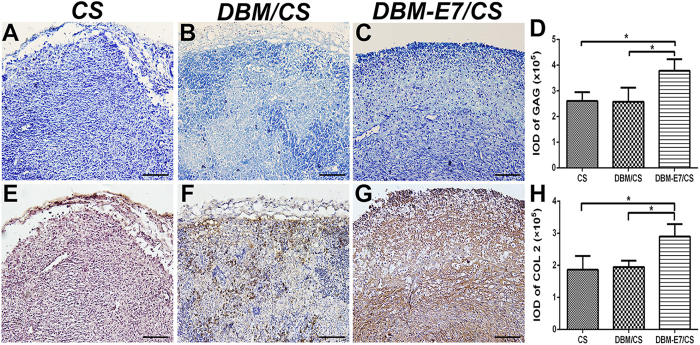
Chondrogenic differentiation of BMMSCs on different scaffolds *in vivo*. (**A–C**) Toluidine blue staining was used to evaluate the production of GAG in CS, DBM/CS, and DBM-E7/CS scaffolds (scale bar = 100 μm). (**D**) IOD analysis of GAG products in the three scaffold groups (*n* = 5, *p < 0.05). (**E–G**) COL2 production was evaluated using IHC staining (scale bar = 100 μm). (**H**) IOD analysis of COL2 in the three scaffold groups (*n* = 5, *p < 0.05).

**Figure 9:**
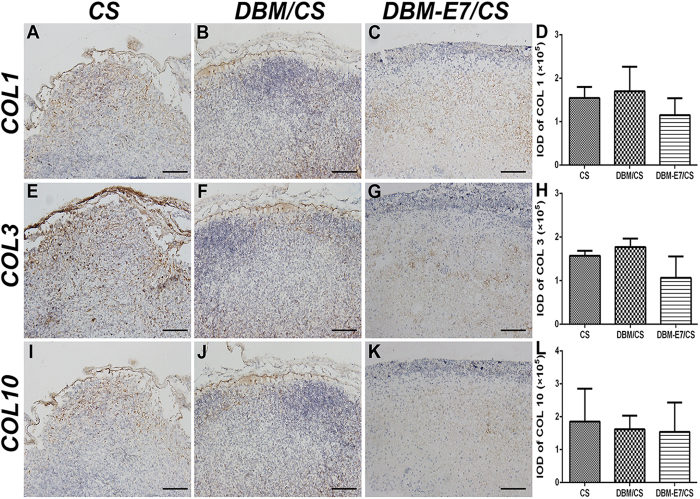
Osteogenesis, hypertrophic scarring, and hypertrophy assessment of the regenerated tissue. (**A–D**) COL1 production was evaluated for osteogenesis. (**E–H**) COL3 production was assessed for hypertrophic scarring. (**I–L**) COL10 was tested for hypertrophy of BMMSCs on different scaffolds by IHC staining. Bar = 100 μm, *n* = 5, p > 0.05.

The three scaffolds were implanted into the fossa iliaca subcutaneous region of the athymic nude mice for four weeks to investigate their biocompatibility and chondrogenesis ability *in vivo*. The animal experiments showed that all types of scaffolds had favorable biocompatibility and demonstrated capacity to regenerate translucent and cartilage-like tissue (Fig. 7A). We observed that the neotissue of the DBM-E7/CS group was larger, heavier, more translucent and callous than that of the DBM/CS and CS groups (Fig. 7B,C). H&E staining indicated a well-defined construct of transplanted cells in the DBM-E7/CS group (Fig. 7F), whereas cells aggregated in the CS group (Fig. 7D) and a heterogeneous construct formed in the DBM/CS group (Fig. 7E). These findings were consistent with the different structures of scaffolds. Cartilaginous ECM was present in all types of scaffolds with positive toluidine blue staining (Fig. 8A–C), and the integrated optical density (IOD) of the DBM-E7/CS group was significantly higher than that of the DBM/CS or CS groups (Fig. 8D). IHC staining of COL2 was analyzed using IPP 6.0 software to determine the chondrogenic differentiation capacity of the three groups. The results indicated positiv COL2 in all groups (Fig. 8E–G), while the content of COL2 of the DBM-E7/CS group was higher than that of the DBM/CS and CS groups (Fig. 8H). The results of IHC staining of COL1 (Fig. 9A–C), COL3 (Fig. 9E–G), and COL10 (Fig. 9I–K) were negative in all types of scaffolds, and the IOD analyses of COL1, COL3, and COL10 showed no difference among the three scaffold groups (Fig. 9D,H,L), indicating negative osteogenesis, hypertrophic scarring, and hypertrophy of the three scaffold groups. These results were consistent with the findings of RT-PCR.

## Discussion

Articular cartilage defect caused by trauma or degenerative pathology is major challenge because of the poor intrinsic healing potential of cartilage. Many attempts including bone marrow stimulation technique, allograft or autograft transplantation, tissue engineering, and autologous chondrocyte implantation procedures have been conducted to repair cartilage defects^27^,^28^,^29^. However, no techniques in the literature have been reported to be able to regenerate functional cartilage similar to the quality of native cartilage. Most of the current available techniques regenerated fibrocartilage tissue with biomechanical properties inferior to hyaline articular cartilage. How to enhance the quality of cartilaginous-regenerated tissue has been the focus of research for decades. Stem cell-based cartilage tissue engineering was believed to be an effective approach^30^.

BMMSCs, which can be conveniently harvested and mobilized by microfracture technique in surgery, provide a promising seed cell source for cartilage regeneration^31^,^32^. However, the amount of BMMSCs in bone marrow stimulated by microfracture is limited such that cell homing techniques have increased the interest of researchers in sports medicine^33^. In our previous study, we identified a BMMSCs affinity peptide named E7 using phage display technology which is able to promote specific BMMSCs homing in cartilage tissue engineering^23^. Microfracture combined with biomaterial scaffolds modified with E7 peptide significantly improved chondrogenic differentiation thanks to the increased amount of BMMSCs for cartilage regeneration. In the current study, we constructed a functional unit of DBM-E7 with highly specific BMMSCs recruiting ability as E7 peptide conjugated with DBM particles. Confocal microscopic images in this study provided evidence that the DBM-E7 particles exhibited better initial adhesion of BMMSCs, which was beneficial for cell growth. The results of CCK-8 test and DNA content analysis also supported the aforementioned conclusion.

Scaffolds is also an important element for cartilage repair and can be applied to enhance cell adhesion and proliferation to produce a matrix and create more complex tissues^34^. Although synthetic materials can be constructed with desirable biomechanical properties, appropriate porosity and biocompatibility, their poor hydrophilicity, unamiable degradation products, and unmatched degradation rate have limited their clinical application^35^,^36^. Natural materials have been widely applied in cartilage tissue engineering because of their excellent biocompatibility, desirable biodegradability, absence of toxic by-products, and amicable cell support microenvironment^37^. CS hydrogel and DBM are two of the most commonly used biomaterials for cartilage repair^38^,^39^. CS hydrogel has adequate porosity and biocompatibility, whereas DBM has notable superior of biomechanical properties. However, poor mechanical properties of CS hydrogel, and inadequate porosity of DBM restricted their clinical application. In the current work, we fabricated a composite scaffold with CS hydrogel and DBM, which was supposed to combine the advantages of CS and DBM and minimize their limitations. The results found that the composite DBM-E7/CS scaffold demonstrated equivalent porosity with CS as well as favorable mechanical properties.

In this study, we ground the whole DBM pieces into particles in order to increase the surface-to-volume ratio, which could enhance the conjugation rate of E7 peptide to DBM and as a result increase the cell adhesive ability of the DBM-E7 particles. Presumably, after adhesion of sufficient BMMSCs on DBM-E7 particles, cell proliferation and gradual accumulation of ECM surrounding the DBM-E7 particles will form an integrated structure of regenerated tissue. The H&E staining results of the neo-tissue generated by DBM-E7/CS scaffold verified our presumption.

According to the literatures, biomaterials can regulate cell growth, proliferation, differentiation, and matrix remodeling^11^,^22^,^40^. The results of CCK-8 test and DNA content analysis in this study confirmed the superiority of DBM-E7/CS scaffold in cell growth and proliferation. The hyaline cartilage-specific gene expression of ACAN and COL2 demonstrated that DBM-E7/CS scaffold promoted chondrogenesis of BMMSCs in producing hyaline-like matrix with its 3D cell support system. Gross observation, histological examination, and matrix staining assessment of chondrogenic tissue generated in nude mice further confirmed the DBM-E7/CS scaffold’s capacity to promote the chondrogenic differentiation of BMMSCs.

Our study had several limitations. Firstly, the whole DBM piece was ground to particles using a Mixer Mill MM 400, and the DBM particles were obtained by sieving. The diameters of DBM particles ranged from 100 μm to 800 μm with different shapes. Standardized preparation of DBM particles might be more desirable because the unequal distribution of DBM particles blended in hydrogel may influence the mechanical properties of scaffolds and cell proliferation. Secondly, the chondrogenic ability of scaffolds was tested in nude mice, and it might be difficult to generalize the conclusions to human articular cartilage repair.

In clinical settings, articular cartilage defects are usually irregularly shaped with different sizes^41^. Most of the currently available biomaterial scaffolds, except for liquid biomaterials, have poor moldability and thus could not fully fill the defects. We proposed a method of combining solid biomatrix particles and hydrogel to integrate the advantages of both CS and DBM. The composite scaffold, modified with the E7 peptide or not, is promising for the repair of irregular cartilage defects because in this study both the DBM-E7/CS and the DBM/CS scaffold exhibited favorable moldability. In the current study, the DBM-E7/CS scaffold exhibited superior chondrogenic capacity *in vitro* and *in vivo*. However, further investigations utilizing these scaffolds in articular cartilage defect of animal models should be conducted to determine the chondrogenesis of scaffolds under a more complex biomechanical environment.

## Materials and Methods

All animal experimental protocols were approved by the Animal Care and Use Committee of Peking University and were in compliance with the Guide for the Care and Use of Laboratory Animals (National Academies Press, National Institutes of Health Publication No. 85-23, revised 1996). The methods were carried out in accordance with the approved guidelines.

## Preparation of Scaffolds

### Preparation of DBM and DBM-E7 particles

DBM was made from the shaft of rabbit femur and tibia according to our previous study^42^. Briefly, the femur and tibia specimens were trimmed and immersed in ethylene diamine tetraacetic acid (EDTA) solution (0.5 M, pH = 8.3) at 4 °C for two weeks, and the solution was replaced every three days. The replaced EDTA solution was analyzed by atomic absorption spectrophotometry to track the demineralization process, and a radiographic check was used to ensure complete demineralization of the specimens. The completely demineralized femur and tibia specimens were placed on the adapters and shaken for 3 min at 28 Hz in a Mixer Mill MM 400 (Retsch Technology, Haan, Germany) after immersion in liquid nitrogen for 5 min. DBM particles with the size of 100 μm to 800 μm was obtained by sieving. The E7 peptides were commercially obtained from Scilight-Peptide Inc., and the conjugation procedures of peptides to DBM were performed according to a previously described method with some modifications^23^,^24^. In brief, 50 mg of DBM particles were immersed in 1 mL of 10% (w/v) 1,6-hexanediamine solution for 1 h at 37 °C. Subsequently, the particles were gently washed twice with ultrapure water and soaked in 400 μL of sulfo-SMCC (Thermo Fisher Scientific Inc., Rockford, IL, USA) solution (2 mg/mL) for 1 h at room temperature. The DBM particles were then incubated in 400 μL of E7 peptide solution (0.1 mg/mL) for 24 h at 4 °C. Ultimately, the DBM-E7 and DBM particles were freeze-dried and stored at −20 °C before use.

### Preparation of CS, DBM/CS, and DBM-E7/CS scaffolds

CS hydrogel was made according to our previous study with slight modifications^15^. In brief, 1 mL of pre-cooled β-glycerophosphate solution (GP; Sigma, St. Louis, MO, USA) was dropped into 2.5 wt% CS solution to create a final CS–GP solution (pH 7.1–7.2). All procedures for preparing this solution were performed on ice to prevent premature gelation. About 100 mg of DBM or DBM-E7 particles was then added into 3 mL of CS hydrogel and blended to form DBM/CS and DBM-E7/CS scaffolds. CS hydrogel, DBM/CS and DBM-E7/CS were separately perfused into a container (diameter of 5 mm and height of 4 mm), freeze-dried, stored at −20 °C, and sterilized by cobalt-60 irradiation before use.

### Characterization of the scaffolds

#### Characterization of DBM-E7 particles

SEM and confocal microscopy images were used to analyze the changes in the properties of DBM before/after peptide conjugation. In brief, DBM and DBM-E7 particles were observed under an S-4800 SEM (Hitachi Ltd, Tokyo, Japan) after vacuum coating with gold in a high-vacuum gold sputter coater. DBM-E7 particles labeled with rhodamine were observed under a Leica TCS-SP8 confocal microscope (Leica, Nussloch, Germany) according to the manufacturer’s protocol. The amount of immobilized peptide was measured using fluorescence assay^16^. DBM-E7 particles were enzymolysized in pepsin solution (1 mg/mL in 0.01 M HCl) after weighing. This DBM digestive solution (20 mg of DBM particles in 1 mL of pepsin solution) was stirred at room temperature for 7 days until no pieces of matrix remained. Fluorescence intensities were measured at 490 nm for excitation and 525 nm for emission using a Varioskan Flash reader (Thermo Fisher Scientific Inc., Rockford, IL, USA). The E7 peptide concentration in the solution was calculated using an E7 peptide standard curve (R^2^ = 0.9935).

### SEM characterization of the scaffolds

The surface and cross-sectional morphologies of the scaffolds were observed under an S-4800 scanning electron microscope (Hitachi Ltd, Tokyo, Japan) operated at an acceleration voltage of 15.0 kV. The scaffolds were vacuum coated with a 5 nm layer of gold in a high-vacuum gold sputter coater after freeze-dried. The images (*n* = 5) were analyzed for pore size and IPP 6.0 software (Media Cybernetics, Bethesda).

### ESR and degradation ratio *in vitro*

Scaffold samples (*n* = 5) were immersed in PBS (pH 7.4) at 37 °C at different interval times, and the swollen scaffold specimens were removed and immediately weighed using a microbalance after removing the excess of water on the surfaces with filter papers. ESR was calculated as follows: ESR = (Ws−Wd)/Wd, where Ws and Wd are the weights of the scaffolds in water-swollen form and at dry state, respectively^15^. To test the *in vitro* degradation ratio, scaffold specimens (*n* = 5) were incubated in PBS (pH 7.4) at 37 °C overnight and then weighed with a microbalance as W_0_. At different intervals, samples were removed and immediately weighed with a microbalance after the excess of water on the surface was removed (Wt). The degradation ratio of scaffolds was calculated as: (W_0_ − Wt)/W_0_ × 100%.

### Mechanical testing

The stress–strain curves and elastic modulus of scaffolds were obtained according to our previous study^15^. Scaffolds (*n* = 3) were tested using a versatile biomechanical system (MTS Systems Corp., Eden Prairie, MN, USA). Scaffold specimens were placed on a test plate at moist state, and a preload of 0.01 N with a 10 N cell was applied. The scaffolds were then compressed at a ramp speed of 0.05 mm s^−1^ until reaching 50% strain. Compressive force and moldability data of the scaffold were collected at 2 Hz using Test Works 4 software (MTS Systems Corp.). The elastic modulus was determined from the slope of the linear portion of the stress–strain curve for all scaffolds.

### Cell cultures and characteristic identification of BMMSCs

BMMSCs were harvested from the distal femur of the 100 g Sprague–Dawley (SD) rats according to previous reports in our laboratory^24^. The primary BMMSCs were incubated at 37 °C with 5% humidified CO_2_ after they were harvested from the bone marrow. After being cultured for 4–5 days, the cells reached confluence and were defined as passage 0 (P0). BMMSCs at passage 3 were used in subsequent experiments. Flow cytometry was used to assess the specific cell surface antigen markers of BMMSCs. Positive markers consisted of CD44 (ab112179), CD73 (ab175396), CD90 (ab225), and CD105 (ab156756), whereas negative markers consisted of CD34 (ab187284) and CD45 (ab10558) (Abcam Inc., Cambridge, MA, USA). A tri-lineage differentiation experiment toward osteogenesis, adipogenesis, and chondrogenesis was also performed to identify the multiple differentiation potential of BMMSCs. The BMMSCs of P3 were incubated in a six-well plate at a density of 10^5^ cells per well with rat BMMSC osteogenic, adipogenic, and chondrogenic differentiation medium (Cyagen Biosciences Inc., Sunnyvale, CA, USA). Alizarin red, oil red O, and toluidine blue staining were used to assess the osteogenesis, adipogenesis, and chondrogenesis of BMMSCs after two weeks of culture, respectively.

### Cell seeding and culture on scaffolds

The BMMSCs of P3 from SD rats were seeded onto the lyophilized CS, DBM/CS, and DBM-E7/CS scaffolds, respectively. In brief, BMMSCs were collected and resuspended at a density of 10^7^ cells mL^−1^, after which 100 μL of this cell suspension (10^6^ cells) was dropped into each scaffold and incubated at 37 °C for 1 h for cell adhesion. Scaffolds seeded with BMMSCs were then cultured in 2 mL of DMEM supplemented with 10% fetal bovine serum for proliferation or cultured in 2 mL of chondrogenic differentiation medium for chondrogenesis with a replacement of medium every 3 days.

### Confocal microscopy

The cell distribution and morphology of BMMSCs seeded on scaffolds were observed under confocal microscopy according to our previous study^43^. The scaffold samples with BMMSCs were washed with PBS for three times and incubated with 0.1% (w/v) AO for 5 min. AO staining images of scaffolds were then obtained using a confocal laser scanning microscope (Leica SP2 inverted microscope; Leica, Mannheim, Germany) equipped with 488 nm lasers. The BMMSCs grown on the three scaffold groups were uniformly stained with green (DNA, 515–545 nm) and red fluorescence (RNA, 590–620 nm). The number of BMMSCs was determined by counting five random fields per scaffold under the confocal laser scanning microscope at ×25 magnification.

### *In vitro* proliferation and matrix formation

A Cell Counting Kit-8 assay (CCK-8; Dojindo Laboratories, Kamimashiki Gun, Kumamoto, Japan) was used to quantify BMMSC proliferation on scaffolds (*n* = 3) *in vitro*. In brief, 200 μL of CCK-8 solution was added to the medium and incubated at 37 °C for 4 h according to the manufacturer’s protocols. The OD was then measured at 450 nm using a Varioskan Flash instrument (Thermo Scientific, Wyman Street Waltham, MA, USA).

The scaffolds seeded with BMMSCs (*n* = 3 in each group at each time point) were weighed with a microbalance and digested in a pre-prepared papain solution (Sigma) at 60 °C overnight. The DNA content of each scaffold was measured by fluorometric assay. The mixture of 20 μL of digestion in each scaffold specimen and 200 μL of Hoechst 33258 working solution (2 μg mL^−1^) was incubated at 37 °C for 1 h. The fluorescence intensities were then measured at 360 and 460 nm for excitation and emission, respectively. The DNA content was obtained according to a standard curve of calf thymus DNA (Sigma).

1,9-Dimethylmethylene blue (DMMB; Sigma) dye-binding assay and enzyme-linked immunoabsorbent assay kit (ELISA; Cloud-Clone Corp., Houston, TX, USA) were used to quantify the sulfated GAG (*n* = 3) and alkaline phosphatase (ALP) (*n* = 3) contents, respectively. About 20 μL of digestion in each scaffold specimen was mixed with 200 μL of DMMB reagent or 200 μL of 0.1 M Tris (pH 7.4) containing 1% Triton X-100 and 5 mM MgCl_2_. The absorbance of GAG and ALP production were measured on a Varioskan Flash instrument at 525 and 460 nm, respectively. The GAG content was determined according to a standard curve based on chondroitin 6-sulfate from shark (Sigma), and ALP production was presented as a ratio of the ALP concentration normalized by the DNA content.

### Cartilage-specific gene expression analysis *in vitro*

Real-time polymerase chain reaction (RT-PCR) analysis was performed using an ABI 7300 RT-PCR system (Applied Biosystems, Foster City, CA, USA) with SYBR Green PCR Master Mix (Toyobo, Osaka, Japan) to determine the gene expression of Aggrecan (ACAN) and type II collagen (COL2) for chondrogenesis and type I collagen (COL1) and ALP for osteogenesis in different scaffolds (*n* = 3 in each group at each time point) after incubation for 7 and 14 days. The PCR primers are presented as follows: ACAN forward primer: 5′-CATTCGCACGGGAGCAGCCA-3′; reverse primer: 3′-TGGGGTCCGTGGGCTCACAA-5′; COL2 forward primer: 5′-CACCGCTAACGTCCAGATGAC-3′; reverse primer: 3′-GGAAGGCGTGAGGTCTTCTGT-5′; COL1 forward primer: 5′-CTGCCCCTCGCAGGGGTTTG-3′; reverse primer: 3′-GCCTGCACATGTGTGGCCGA-5′; ALP forward primer: 5′-GCTACACCACAACACGGGCGA-3′; reverse primer: 3′-TCCAAATGCTGATGAGGTCCA-5′; 18s RNA forward primer: 5′-GTAACCCGTTGAACCCCATT-3′; reverse primer: 3′-CCATCCAATCGGTAGTAGCG-5′. Moreover, 18s RNA was used as the housekeeping gene. The amplification program of RT-PCR was as follows: 95 °C for 2 min, followed by 40 cycles of 95 °C for 15 s and 60 °C for 1 min. Melting curve analysis was added at the end of the amplification procedure, and the melting curve showed no nonspecific amplification. The relative changes in target gene expression were quantified using the ∆∆Ct method^44^.

### Gross observation, histological assessment, and matrix staining *in vivo*

The three groups of scaffolds seeded with BMMSCs were first cultured with chondrogenic differentiation medium for one week. The specimens were then cut into small equal pieces and subcutaneously implanted into nude mice. Fifteen nude mice were randomly divided into three groups (*n* = 5 in each group). After four weeks of culture, the diameter and weight of the scaffold specimens were measured at wet state after being harvested. The generated tissue was fixed in 4% paraformaldehyde (pH 7.4) for 24 h at room temperature. The specimens were dehydrated in a graded ethanol series and embedded in paraffin. Serial sections (4.5 mm-thick) were sagittally cut and stained with hematoxylin and eosin (H&E) and toluidine blue. Immunohistochemical (IHC) staining was performed with COL2, COL1, COL3, and COL10 antibodies (Abcam, Cambridge, UK) to test chondrogenesis, osteogenesis, hypertrophic scarring, and hypertrophy of scaffolds.

### Statistical analysis

The results were expressed as the mean ± SD and represented at least three independent experiments. The differences between groups were analyzed using one-way ANOVA after testing for homogeneity of variances by SPSS 20 software. Pairwise post hoc tests were performed with LSD multiple comparison procedure. Statistical significance was set at P < 0.05.

## Conclusions

In this study, we established a composite scaffold named DBM-E7/CS by combining the MSCs affinity peptide-modified DBM particles and CS hydrogel. This scaffolds exhibited suitable biomechanical properties, excellent biocompatibility, highly specific BMMSCs recruiting capacity, and favorable chondrogenesis *in vitro* and *in vivo*. The composite DBM-E7/CS scaffold is a promising option for repairing irregularly shaped cartilage defects.

## Additional Information

**How to cite this article**: Meng, Q. *et al.* A composite scaffold of MSC affinity peptide-modified demineralized bone matrix particles and chitosan hydrogel for cartilage regeneration. *Sci. Rep.* **5**, 17802; doi: 10.1038/srep17802 (2015).
